# Time series data with and without anomalies from a continuous distillation plant

**DOI:** 10.1038/s41597-026-08308-7

**Published:** 2026-10-01

**Authors:** Aparna Muraleedharan, Alvaro Ferre, Justus Arweiler, Indra Jungjohann, Fabian Jirasek, Hans Hasse, Jakob Burger

**Affiliations:** 1 Technical University of Munich, Campus Straubing for Biotechnology and Sustainability, Laboratory for Chemical Process Engineering, Uferstraße 53, 94315 Straubing, Germany; 2 Laboratory of Engineering Thermodynamics (LTD), RPTU Kaiserslautern, Erwin-Schrödinger-Straße 44, 67663 Kaiserslautern, Germany

## Abstract

Reliable detection of process anomalies remains a challenge in industrial chemical plants. The ability of machine learning (ML) to recognize patterns has triggered numerous research efforts to apply ML to anomaly detection (AD). Simulation-based benchmarks, such as the Tennessee Eastman process, are widely used to develop and train AD methods. Well-annotated real-world process data, which are crucial for meaningful research advancements, are lacking due to industry-wide proprietary limitations. To overcome this issue, we present an openly accessible dataset of time series generated from an industry-like continuous distillation mini-plant under constant operating conditions. The data generated have different complexities: water runs, a heteroazeotropic separation of n-butanol–water, and a reactive process to produce a fuel additive. Chemical systems, plant setup, and anomalies (whether encountered or manually induced) are described alongside the process data. The complete dataset, including sensor and actuator data, annotations marking anomalies, and other metadata, is available in open access via an online Zenodo repository. It serves as training and testing data for ML-based AD and other data-driven applications.

## Background & Summary

In the chemical industry, continuous processes are essential to optimize efficiency and produce high-quality products on large scales^1,2^. The stability of critical process parameters during plant operation is maintained by control strategies^3^, which counteract disturbances, such as fluctuations in input feeds, external conditions, or within the process. Despite robust control strategies, anomalies (deviations from normal behavior) can still occur^4^. In the present work, we distinguish anomalies from disturbances as follows. Disturbances are variations of the process inputs or environment that occur routinely and that the plant is designed to manage, e.g., fluctuations in the feed flow rate, variations in the cooling-water temperature, or changes in the ambient temperature. The resulting variations in the process variables remain within the range expected at the given operating point and require no external intervention. An anomaly, in contrast, is a deviation of the process behavior from that expected at the given operating point that cannot be explained by such routine variability, is not corrected by the control system alone, and generally requires intervention, e.g., because of a fault in the equipment or instrumentation. Anomalies can indicate potential malfunctions, safety hazards, or inefficiencies that, if left unaddressed, may result in catastrophic failures or substantial economic losses.

This distinction is context-dependent and cannot be made universal. For example, a variation in the product flow rate of  ±30% may be entirely normal in a separation process fed from an upstream batch process, whereas the same variation would indicate a fault in a fully continuous plant. The distinction also depends on the threshold selected for slow drifts, which may be regarded either as normal equipment ageing or as the onset of an anomaly. A survey and analysis by Kister^5^ spanning five decades shows that the anomalies in distillation towers are repetitive and have continued to increase. Plugging and coking dominate the list of anomalies, followed by reboiler level and internal damages. With the industry’s growing dependence on automated systems and advanced technologies, effective, early detection of anomalies using reliable techniques is crucial for maintaining safety, optimizing efficiency, and meeting regulatory requirements^6^.

Even though anomalies are rare, machine learning (ML) methods have proven effective in detecting anomalies from large datasets and reached high accuracies in a wide set of application domains^7–10^. Also in the chemical process industry, which is the domain of the present work, ML-based AD has seen several research developments in the past decade, with new methods and techniques being proposed, such as deep learning or reinforcement learning^11–14^. More recent innovations in ML, such as Generative Adversarial Networks (GANs) and Long Short-Term Memory (LSTM) networks have shown promise in enhancing AD capabilities by effectively modeling complex data patterns, which are inherent in many chemical processes^15^. Although ML methods for AD are being developed at a high pace, the available data sources for training and testing have not kept up. As a rule, process data from the chemical industry is not openly available due to proprietary limitations. This led Downs and Vogel^16^ in 1993 to create an imaginary industrial process, the Tennessee Eastman Process (TEP), and publish a dynamic process model for it.

For three decades, the TEP has continued to serve a broad audience as a robust educational and research tool, applicable to plant-wide control, multi-variable control, optimization, as well as AD^17^. The TEP is a chemical process with five unit operations: a reactor, a product condenser, a vapor-liquid separator, a recycle compressor, and a product stripper. From its simulation, time-series datasets can be produced and are available for download^17,18^. Although the TEP datasets provide a good benchmark for method development, it is crucial to gauge the applicability of the methods in a real-world setting, where the performance of methods trained to perform well with TEP data might be different. For example, we have performed a comprehensive evaluation of deep learning AD methods in the literature using the TEP dataset and concluded that generative model types perform the best^19^. However, when we applied the same methods to time-series data from all three experimental scenarios of the present work, no single method performs consistently best across all datasets, and the ranking depends strongly on the chosen metric and process scenario^20^. Such results emphasize the importance of utilizing real-world data in the development and evaluation of data-driven AD methods.

Publicly available datasets of real-world time series, such as the MIMII, IPAD, or VISION^21–23^, focus on sound, video, or images from industrial machines such as valves and pumps. They contribute to industrial equipment maintenance, but are not typical time series data from chemical plants. Although several studies in the literature^24–27^ actually use real-world data from chemical plants for AD, process optimization, and control applications, none of them have published the data. It is striking that there is still a lack of publicly available chemical process data^28^. Industrial companies often implement measures to anonymize the data (anonymization involves removing or masking identifiable information to protect privacy and confidentiality) when sharing it with other parties. Ideal training datasets are, however, not anonymous^29^ and contain additional metadata or context. Applied to chemical process data, this additional information can include, e.g., the process flow diagram, the piping and instrumentation diagram, property data of the chemicals used, or annotations to anomalies by the operators. Including this information ensures that models can detect realistic issues and that the results are interpretable and reproducible.

In the present work, we supply real-world process data from a continuous distillation plant in our lab. Although operated in an academic environment, the plant is run in an industry-like manner, including a process control system that maintains constant operating conditions for hours or days, depending on the operating point. Distillation is one of the most common unit separations used in the chemical industry, making the data valuable for use in the development of data-driven ML methods. The dataset presented here combines historical operation data from an older project^30^ with novel operating points that were run simply for the sake of data generation. A particular focus of the present work is to provide data for training of AD methods; thus, we provide data with and without anomalies, which are labeled respectively. The scope of the anomalies in the current work is further described in the Usage Notes section. Together with a novel dataset for batch distillation, which we have reported in a separate paper^31^, we have created a solid database for testing and developing ML methods; see, e.g.^32,33^, for initial applications in collaboration with project partners. The resulting dataset comprises around 580 hours of continuous operation under constant operating conditions at several operating points across three distinct scenarios and is released under a CC-BY-4.0 license.

## Methods

### Continuous distillation mini-plant

**Fig. 1:**
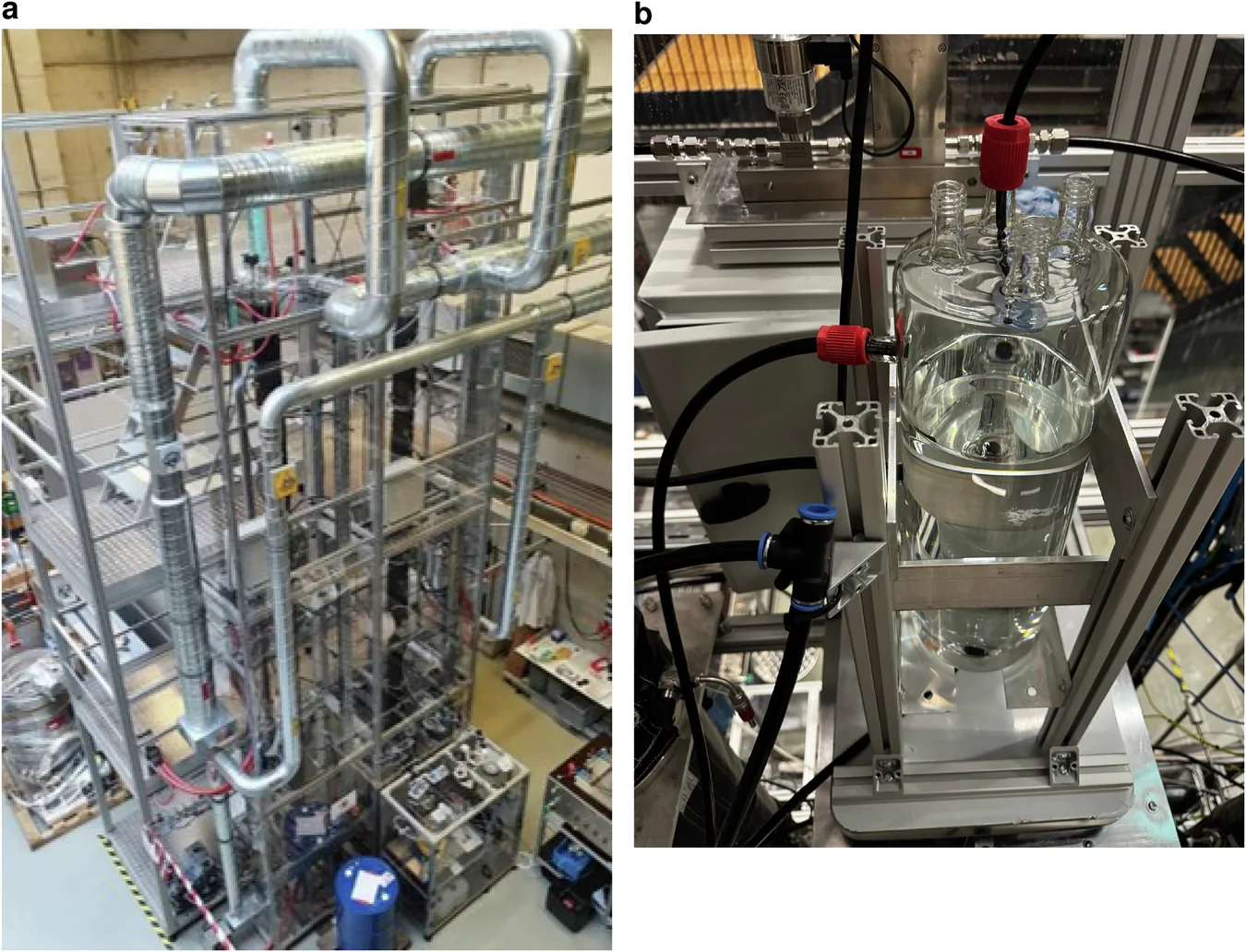
Overview of the continuous distillation plant. **a**, Continuous distillation mini-plant. **b**, Decanter used for liquid–liquid phase separation.

**Fig. 2:**
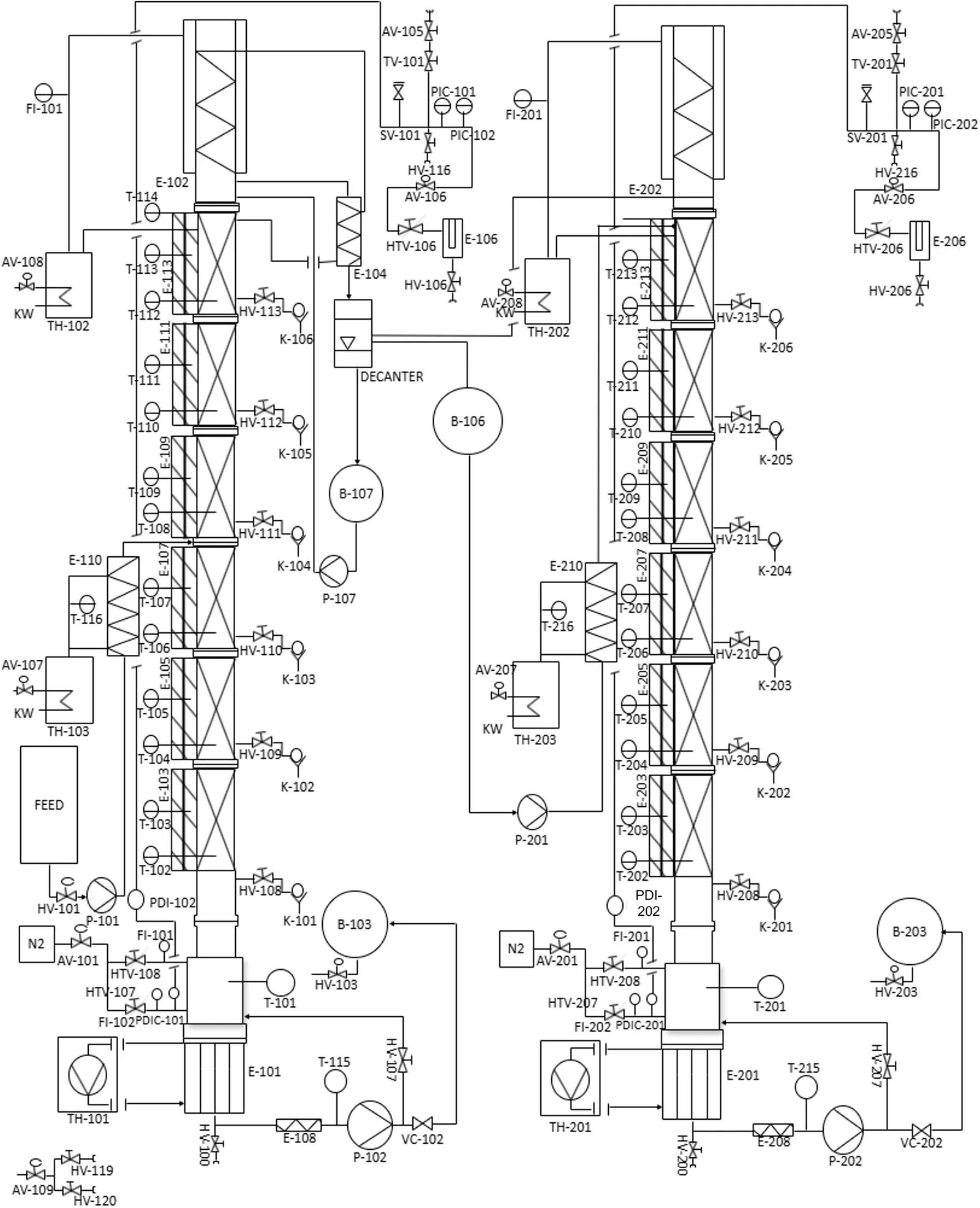
P&ID of the continuous distillation plant for the heteroazeotropic distillation of n-butanol–water.

The mini-plant (shown in Fig. 1) used in the present work was originally erected for the production of poly(oxymethylene) dimethyl ethers (OME_3−5_) by Ferre *et al*.^30^, following a process developed by Schmitz *et al*.^34^. It consisted of a tubular reactor, a membrane separator (DBI Gas and Umwelttechnik), and two distillation columns (Iludest GmbH). These two distillation columns were the object of the present work. We prepared and published the data originally recorded by Ferre *et al*.^35^ and, in addition, carried out further experiments with other chemical systems. For this purpose, the setup was augmented with a decanter and with additional control strategies for heteroazeotropic distillation, which are described in the following section. The plant is described here in the configuration in which the data reported in this work were recorded. Fig. 2 shows a piping and instrumentation diagram (P&ID) of the two columns and the decanter. The first column, C1, shown on the left side of Fig. 2, had a diameter of 70 mm, was made of stainless steel, comprised six sections, and was rated for pressures up to 2 bar (a). The second column, C2, had a diameter of 50 mm, was made of glass, comprised six sections, and was rated for pressures up to 1 bar (a). The cylindrical glass decanter, manufactured in-house, had a total height of 37 cm and a diameter of 13.36 cm. Each section of C1 and C2 was insulated with electric heating jackets (SAF) to minimize heat losses. Both columns were equipped with a partial reboiler and a near-total condenser. Temperature sensors (e.g., T101) were positioned at the reboiler, at the bottom of each column section, and at the top of the column. Sampling valves (e.g., HV208) were installed between the sections and allowed liquid samples to be withdrawn during an experiment.

The head pressure sensors (PIC101, PIC201) recorded the top pressure in the columns. A small flow of an inert gas (nitrogen) was used to control the head pressure. The condensers (E102, E202) used water from the grid and were located at the top of the columns. The distillate from C1 underwent a second cooling process in exchanger E104, operated with a mixture of ethylene glycol and water, which was back-cooled in a cryostat (TH102). Uncondensed gases were collected in the cold traps filled with liquid nitrogen (E106, E206). The reboilers (E101, E201) were shell-and-tube heat exchangers with thermostats (TH101, TH201) heating the oil fed into the shell, while the liquid product evaporated in the tubes. Differential pressure measurement indicators (PDIC101, PDIC201) measured the liquid levels in the reboiler. Both columns were equipped with 3.84 m of structured packing (Sulzer CY).

The feed tank was connected to a membrane pump, which pumped the feed through a heat exchanger (E110), heated using oil from a thermostat (TH103). The bottom products of the columns were cooled in heat exchangers (E108, E208) using grid water at room temperature and drawn using gear pumps (P102, P202). If the feed mixture was heteroazeotropic, a decanter for liquid-liquid phase split was employed as a common reflux drum for both columns. The upper (organic) phase was fed as reflux into C2 with a gear pump (P201) and the lower (aqueous) phase as reflux to C1 with another gear pump (P107). Flow indicators (Coriflow) measured the flow rate of the feed (F104), the distillates from both columns (F102, F103), and the reflux stream to the first column (F101). Weighing scales (A101, A102, A103, A104, A106, A107, and A203) were used to measure the mass of buffer vessels, thereby calculating the mass flow rates of several other streams.

The software LabVIEW v.19 was used to control the operation and record the data of the mini-plant. Overall, the mini-plant had two pressure controllers, three flow controllers, four level controllers, and two temperature controllers. All controllers were PID, except the level controllers in the reboilers, which were P-only and sufficient for a quick, reliable response. The control loops and strategies are described in later sections, as they are scenario-specific. The mini-plant setup generated multivariate time-series data from up to 38 sensors and 11 actuators, depending on the experimental scenario. In addition to the sensor data, concentration analysis data of liquid samples from each experiment were generated via scenario-specific offline analytics, also described in later sections. Fig. 1 gives a combined overview of the continuous distillation plant and the decanter that was added. Table 1 shows the range of operating parameters of the plant. Detailed information regarding the sensors, the associated P&ID labels, the related suppliers, and the precision of these instruments is provided in Tables 2 and 3.

**Table 1:** Specifications of the continuous distillation plant.

Parameter	Lower limit	Upper limit
C1 design pressure/kPa	1	200
C2 design pressure/kPa	2.5	100
Reflux fraction/g. g^−1^	0	1
C1 design temperature/K	293.15	473.15
C2 design temperature/K	293.15	473.15

**Table 2:** Sensor & actuator information.

Equipment	P&ID labels	Model	Manufacturer	Manufacturer specified performance
Flow indicators	F101, F102, F103, F104	Coriolis Miniflow	Bronkhorst	Accuracy ±0.2%
Differential pressure sensor	PDIC101, PDIC102, PDIC201, PDIC202	PD-33XEi	Keller	Accuracy ±0.05%
Feed (Membrane) pump	P101	EXBb G0613SS10A420	Prominent	Reproducibility ±2%
Pressure	PIC101, PIC201	Typ266AST	ABB	Base accuracy ±0.04%
Pumps	P102, P107, P201, P202	1A-A/3-19/D-SS, 1MA-A/6/D-SS	Gather	
Temperature	T101, T102, T104, T106, T108, T110, T112, T114, T115, T116, T201, T202, T204, T206, T208, T210, T212, T215	Pt-100 (GTF103-Ex)	Greisinger	Stability ±0.15 ^°^C
Thermostats	TH101, TH102, TH103, TH201, TH202, TH203	CC-202C, CC304-B, HTS 3, HTS 5	Huber	Stability ±0.02 ^°^C
Weighing scales	A101, A102, A103, A104, A106, A107, A203	DE	Kern	Readability 0.1 g to 120 kg

**Table 3:** P&ID labels and their descriptions.

P&ID label	Description
B101	Feed Tank
B103	Bottoms product tank column 1
B106	Decanter upper phase tank
B107	Decanter lower phase tank
B203	Bottoms product tank column-2
C101 to C106	Column 1 parts 1 to 6
C201 to C206	Column 2 parts 1 to 6
E101, E201	Reboiler heater
E102, E202	Column head
E103, E105, E107, E109, E111, E113	Tower heating mantle column 1
E108, E208	Bottoms product cooler
E110, E210	Feed heater
E203, E205, E207, E209, E211, E213	Tower heating mantle column 2
HV101	Manual draining valve B101
HV103	Manual draining valve B103
HV108, HV109, HV110, HV111, HV112, HV113	Manual sample valve column 1
HV203	Manual draining valve B203
HV208, HV209, HV210, HV211, HV212, HV213	Manual sample valve column 2
P101	Feed pump
P102	Bottoms product pump column 1
P107	Decanter lower phase pump
P201	Decanter upper phase pump
P202	Bottoms product pump column 2
PDIC101, PDI102	Pressure difference indicators column 1
PDIC201, PDI202	Pressure difference indicators column 2
PDIC103, PDIC203 (not shown in the P&ID)	Distillate tank level controllers
T101	Temperature sensor reboiler column 1
T102, T104, T106, T108, T110, T112	Temperature sensor in column 1
T103, T105, T107, T109, T111, T113	Temperature sensors integrated in tower mantle
T114	Head temperature sensor
T115	Temperature sensor in bottoms product line
T116	Temperature sensor in feed line
T201	Temperature sensor reboiler column 2
T202, T204, T206, T208, T210, T212	Temperature sensor in column 2
T203, T205, T207, T209, T211, T213	Temperature sensors integrated in tower mantle
T215	Temperature sensor in the bottom product line

### Scenarios for generating experimental data

Experiments in three scenarios (i.e., three different chemical systems) were conducted to generate continuous time-series data under constant operating conditions at several operating points with varying levels of complexity.

- A: Water runs with only the first column
- B: Separation of the heteroazeotropic mixture of n-butanol–water using both columns and a decanter
- C: Separation of OME_3−5_ from a reactive mixture of water, formaldehyde, methanol, and OME in only the first column

The chemical systems in the three scenarios and their respective operating conditions were chosen based on factors such as process safety, data utility, and relevance to actual industrial applications. Experiments in Scenario A facilitated the remote operation of the plant and enabled safe, unattended overnight operation, consequently producing experimental data (61286 data points, 510.7 hours of operation) over an extended period of 30 days. Scenario B is a typical separation process within the chemical and pharmaceutical sectors, e.g., in biofuel synthesis^36,37^. In Scenario C, chemical reactions occurred inside the column, yielding a reactive distillation, another interesting application example.

**Fig. 3:**
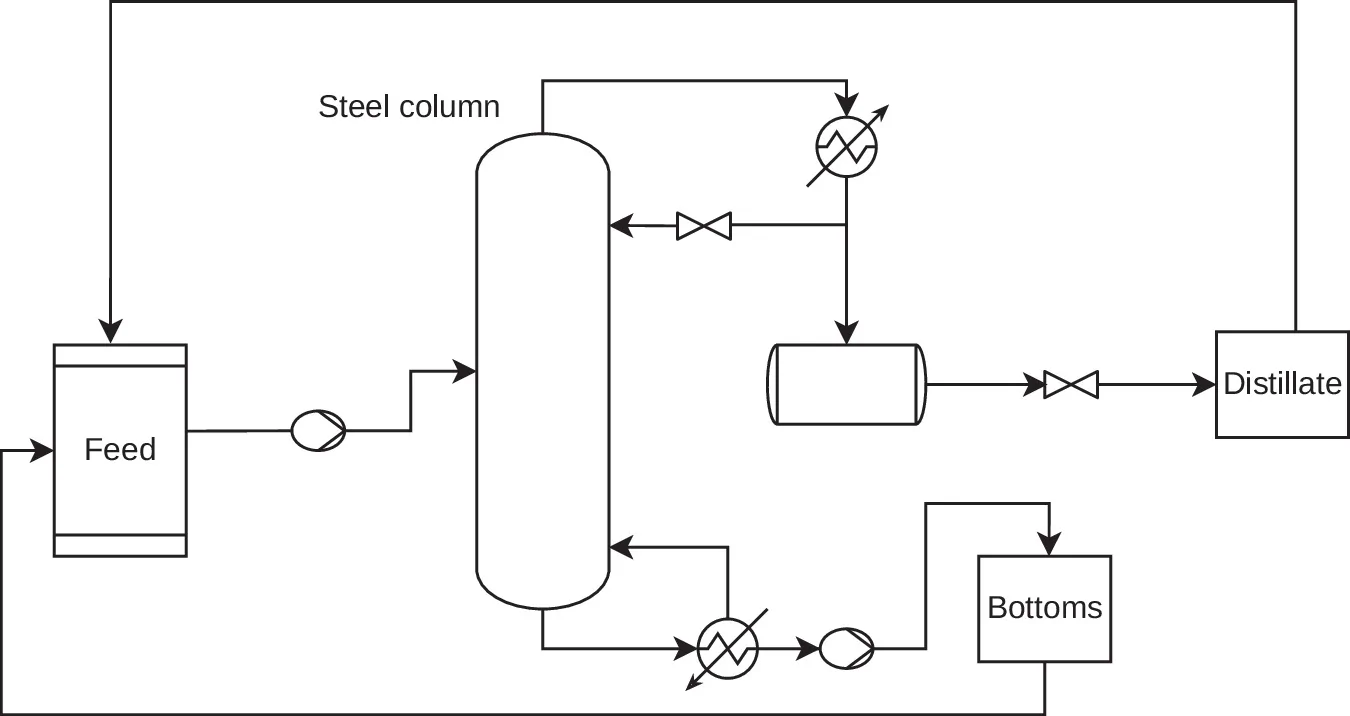
PFD of the continuous distillation plant for experiments with water.

Experiments in Scenario A involved feeding water into C1 at a packing height of approximately 2.5 m (middle of the packing); the distillate and bottom products were collected and recycled back into the feed tank. Constant operating conditions were achieved within one hour of plant startup. The bottom product flow rate, the feed flow rate, and the reflux ratio were controlled. The temperature in the oil thermostat (TH101) for the reboiler was regulated to maintain the reboiler level (PDIC101) using a P-only controller. The head pressure (PIC101) was controlled using a PID controller, and the liquid level in buffer tank B102, which collects the distillate, was maintained by another PID controller (PDIC103). Fig. 3 represents the schematic configuration of the continuous distillation process using water.

Scenario B involved a heteroazeotropic distillation of an n-butanol–water mixture. In several runs with feed compositions ranging from 0.06 g/g to 0.20 g/g n-butanol, the mixture was fed into C1 at a packing height of approximately 2.5 m. The bottom product from C1 was rich in water and the condensed distillate was sub-cooled and fed into the decanter, where it split into two liquid phases. The lower (aqueous) phase was fed back into C1 as the reflux, whereas the upper (organic) phase was fed into C2 at the top of the packing. The bottom product from C2 was rich in n-butanol, and the condensed distillate was subcooled before being fed into the decanter. Both columns operated at atmospheric pressure. Constant operating conditions were achieved within 3 hours of plant startup. Figure 2 shows the plant configuration used for this scenario. The set of recorded sensors was not identical at every operating point. Additional flow meters were installed during the n-butanol–water campaigns, so the earlier operating points contain fewer flow measurements than the later ones.

The control strategy proposed by Luyben^38^ was used here: the oil in the reboiler thermostats (TH101, TH201) was maintained at 413.15 K in C1 and 403.15 K in C2. The reboiler liquid levels (PDIC101, PDIC201) and the decanter overflow buffer tank levels (B106, B107) were maintained using level controllers by manipulating the flow through the respective pumps (P102, P202, P107, and P201). Seventeen liquid samples from C1, C2, the decanter, and the feed tank were collected and analyzed during each experiment. Since the n-butanol–water mixture is a heteroazeotrope, 1-propanol was added to the collected liquid samples to ensure a homogeneous mixture at room temperature. The mass fraction of n-butanol in the samples was measured using gas chromatography (GC) with tetrahydrofuran (THF) as the internal standard. The gas chromatograph employed was a Thermo Fisher Scientific Trace 1610 (Flame Ionization Detector (FID), detector temperature 523.15 K, injection temperature 503.15 K, carrier gas: Helium, split flow: 150 mL min^−1^, Restek Rtx-Wax column). Pure n-butanol and 1-propanol were used for calibration. Each measurement was repeated twice, and the two repeat measurements differed by less than 0.05% (absolute) in n-butanol mass fraction on average. The GC method was evaluated to have a relative error of 1.8%, calculated using known n-butanol samples. Karl Fischer titration was used to determine the water mass fractions in samples with less than 0.21 g/g water. Karl Fischer titration was also repeated twice for each sample, and the absolute difference between the two measurements was less than 1% of the water mass fraction on average. The uncertainty of Karl Fischer titration was evaluated by measuring samples with a known amount of water, and the relative error was found to be 1.7%. Karl Fischer was preferred over GC when measurements from both methods were available.

In Scenario C, the experiments with the reactive OME system as described by Ferre *et al*.^35^, involved feeding a methanolic formaldehyde solution into a tubular reactor filled with a catalyst (Amberlyst 46)^39^. The reactor product was then fed into C1 at a packing height of approximately 2.5 m. The main objective of C1 was to produce OME_3−5_ with minimal formaldehyde impurities by separating formaldehyde and other components as the distillate from OME_3−5_ as the bottom product. The reactor and catalyst loading are described in detail by Ferre *et al*.^35^ and were not varied here. Constant operating conditions were achieved within 4 to 5 hours of plant startup. In those experiments, the control strategy used was the same as that employed in the experiments with water in Scenario A: The reboiler oil temperature in thermostat TH101 was manipulated to maintain the reboiler level (PDIC101) using a P-only controller, the head pressure (PIC101) was controlled using a PID controller, and the distillate liquid level (PDIC103) was also controlled using a PID controller. In addition, the bottom product flow rate, feed flow rate and the reflux ratio were set. Analytical methods such as GC, Karl Fischer titration, and sodium sulfite titration have been employed here to detect the concentration of different components; more details are provided in Ferre *et al*.^35^. In all 3 scenarios, we chose P-only control of the reboiler level because a small permanent offset in the level is acceptable, and the loop responds quickly without integral windup. Details regarding operating points for each scenario, such as feed flow rate, concentration, and operating head pressure are provided in Supplementary Table 1.

### Generation and labeling of anomalies

Table 4 lists the observed anomalies in our continuous distillation plant and the affected sensors. Many anomalies were manually triggered. For example, a clogging in the feed pipeline was manually triggered by closing a valve in the feed pipeline.

**Table 4:** Distillation column anomalies relevant to our mini-plant.

Anomaly category	Affected sensors or actuators	Specific location
Anomalous operation incidents	TH101, TH201, TH103, TH203	Reboiler overheating, Condenser malfunction
	PIC101, PIC201	Pressure build-up
	PIC101, PIC201	Pressure loss
Clogging	A107, A106, PDIC101, PDIC201	Pipes, outlets, packing
	A103, A203	Pipes, outlets, packing
Condenser control problems	TH102, TH202, AV108, AV208	Cooling water line
Condenser malfunctions	TH102, TH202	Coolant liquid refill
Faulty instruments	PDIC101, PDIC201	Level sensor measurements
Reboiler level instability	PDIC101, PDIC201	Reboiler
Operating error	—	LabVIEW crash
Pressure control problems	PIC101, AV105, AV106	Inert gas flow

The most common anomalies were clogging, reboiler level instability, overheating, faulty instruments, pressure control problems, and operating system crashes. Table 5 lists the methods used to induce the anomalies (excluding the naturally occurring anomalies). For all anomalies, we have provided the time frame of their occurrence and the affected sensor in the metadata file. We have also provided details on when the anomaly was induced, when it became visible in the data, and when the system recovery was performed, where available.

**Table 5:** Inducing anomalies in the mini-plant.

Anomaly category (Specific location)	Strategy
Anomalous operation incidents (Reboiler overheating)	Gradually increased the thermostat (TH101, TH201) temperature by 10 K and, when the reboiler level (PDIC101 at 1.1 mbar or PDIC201 at 0.35 mbar) fell below the minimum threshold, reset to the constant operating value.
Condenser control (Cooling water line)	Gradually raised the thermostat (TH102 or TH202) by 10 K or switched off the automatic cooling water valve (AV108), then reset once the distillate flow rate fell below a safe threshold.
Condenser malfunction (Coolant liquid refill)	Not refilling thermostat coolant liquid timely, led to insufficient cooling.
Clogging (Pipes)	Partially closed the feed manual valve and reopened once the reboiler level (PDIC101 or PDIC201) dropped below a safe threshold; similarly, partially closed the bottoms manual valve and reopened it when the level rose above the safe threshold (PDIC101: 2 mbar, PDIC201: 1 mbar).
Faulty instruments (Level sensor measurements)	Loosened the pipes to the level devices (PDIC101/PDIC201) or altered RS232 adapter settings to simulate faults.
Reboiler level instability (Reboiler)	Switched off TH101/TH201 until the reboiler level (PDIC101/PDIC201) increased above a safe threshold.
Pressure control problems (Inert gas flow)	Manually opened/closed the pressure release valve, then reset to the original position once pressure exceeded the threshold (PIC101 at 1.5 bar (a)).

### Data processing

Sensor data were recorded every 30 seconds. For all scenarios, the startup and shutdown phases were removed to retain only data recorded under constant operating conditions, apart from routine disturbances, controller actions, and measurement variability. For Scenarios A & C, the timestamps were left as recorded. For Scenario B with two distillation columns, technical reasons necessitated the use of two separate data acquisition systems that could not be synced during the recording. To provide a consistent dataset for training AD methods, we rounded the timestamps to the nearest 30s. In some early runs, one column produced readings every 20 seconds; in those cases, the values within the same 30-second window (e.g., between 10:20:00 and 10:20:30) were averaged and stored at the beginning of the window (e.g., 10:20:00). The processing was performed with a custom Python script (v1.0, archived at https://doi.org/10.5281/zenodo.21814763); see the Code Availability section.

## Data Records

**Fig. 4:**
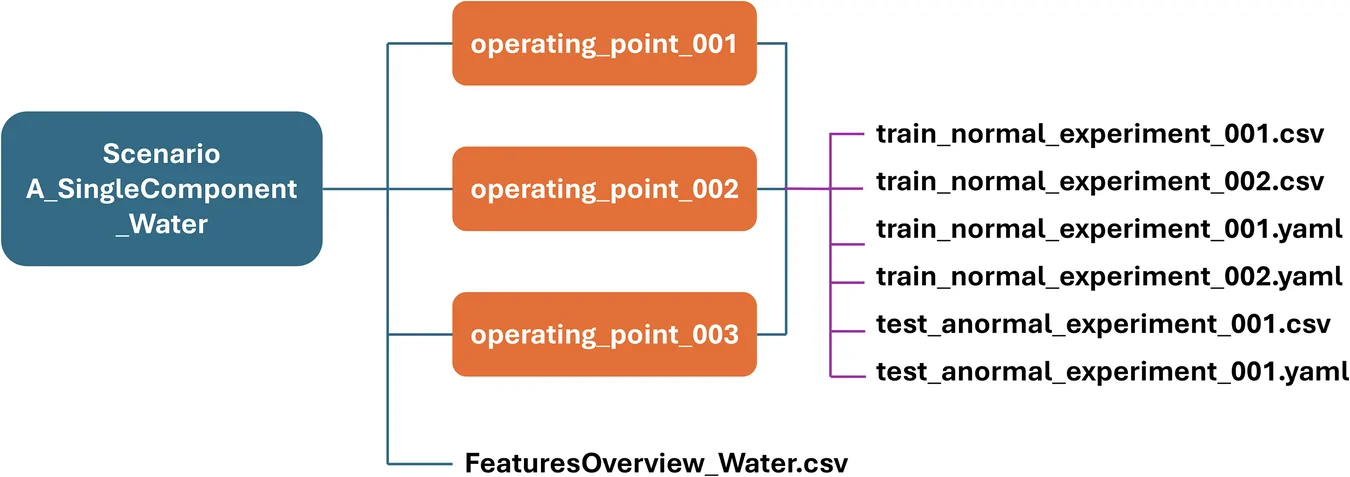
Data repository folder structure sample.

The dataset is available at Zenodo^40^. An example of the folder layout is shown in Fig. 4. Each scenario (e.g., Scenario A_SingleComponent_Water) has operating points grouped by key operating parameters such as pressure, feed flow rate, and feed composition (e.g., operating_point_001). Within each operating point, data are provided for both anomalous (e.g., test_anormal_experiment_001.csv) and non-anomalous experiments (e.g., train_normal_experiment_001.csv). Each scenario includes a sensor description file (e.g., FeaturesOverview_Water.csv), and for each operating point, metadata for individual experiments is provided, including information on operating pressure, feed concentration, feed flow rate, and reflux ratio (e.g., test_anormal_experiment_001.yaml).

Each row is one 30-second time step. Time is the clock time of the reading, HH:MM:SS, with no date; rows within a file run consecutively. Temperatures are in ^∘^C, at the positions marked in Fig. 2: T101-T116 in C1, T201-T214 in C2. PDIC101 and PDIC201 are the differential pressures across the reboilers (in mbar), which we use as a measure of reboiler levels; PDIC102 and PDIC202 are the differential pressures across the packing. The differential pressures across the packing appear as PDIC102 and PDIC202 in the data files; they are shown as PDI-102 and PDI-202 in Fig. 2, as no control loop was configured for them in the experiments reported here. Head pressures are PIC101 and PIC201 in mbar.

Flow rates are in kg h^−1^ but come from two different measurements. The F columns in kg h^−1^ are Coriolis flow meters - F104 is the feed to C1, F102 and F103 are the distillates of C1 and C2, and F101 is the reflux to C1. The M columns are instead derived by differentiating the balances under the buffer vessels, whose masses in kg are shown in the A columns; M103 and M203 are the bottoms of C1 and C2, in kg h^−1^. In addition to the measured variables, the files contain the set point of each control loop, the thermostat set temperatures in ^∘^C (TH columns), the pump power settings as a percentage of full power (P201), and the states of the automatic valves, level sensors, flow pulse sensors, pumps, and thermostats as True or False (AV, PS, FS, THxxx_Status, P102, P202 columns).

For each run and at each time step, separate label columns are provided to describe the different anomaly types and their levels of detail. The “common” labels give a simple binary indication of whether an anomaly is present, while the “advanced” labels show how the anomaly develops over time, from its cause to its final effect.

- **Label (common/hard fault):** 0 or 1, indicating when a severe anomaly is present, from the beginning of its cause to the end of its effect.
- **Label (common/soft fault):** 0 or 1, showing the presence of a mild or transient anomaly.
- **Label (common/hard and soft):** 0 or 1, showing the presence of an anomaly related to the process, including severe or transient anomalies.
- **Label (common/controller fault):** 0 or 1, showing the presence of an anomaly related to controller behavior rather than the process itself.
- **Label (common/all):** 0 or 1, marking the occurrence of any anomaly, regardless of severity, over the entire duration from cause to effect.
- **Label (advanced/hard fault):** 0, 1, 2, or 3, indicating the phase of a severe anomaly.
- **Label (advanced/soft fault):** 0, 1, 2, or 3, showing the phase of a mild or transient anomaly.
- **Label (advanced/controller fault):** 0, 1, 2, or 3, describing the phase of an anomaly related to controller behavior rather than the process itself.

For naturally occurring anomalies, we use binary labels: 0 for normal data points and 1 for anomalous ones. For manually induced anomalies, there is a more detailed integer label scheme: 0 for normal data points, 1 for when an anomaly is induced but not yet visible in the data, 2 for when an anomaly is visible in the data, and 3 for the recovery phase when the cause of the anomaly is removed but the effects are still visible in the data. The sensor description file supplied with each scenario lists the sensors recorded. The YAML file for each run gives the operating pressure, feed concentration, feed flow rate, and reflux ratio. For runs with anomalies, it also provides the start and end times of each anomaly, the affected sensors, and the cause.

## Data Overview

Experiments involving water generated time-series data over a 30-day period. Each experiment with n-butanol–water typically lasted about 10 hours, with some extending overnight. Similarly, experiments using OME, as reported by Ferre *et al*.^35^, also averaged 10 hours, including a few overnight sessions. Table 6 shows the statistics regarding the number of experiments, total duration of experiments, and the number of anomaly-free and anomaly runs. The time series data and concentration profiles can be visualized using a graphical, easy-to-use web interface built on a Streamlit-based application^41^. Additional details on this web interface are provided in the Usage Notes section.

**Table 6:** Overview of experimental scenarios and extent of data collected.

Scenario	Total runs	Normal runs	Anomalous runs	Total duration of constant operating conditions (hrs)	Time points
Scenario A	12	4	8	510.7	61286
Scenario B	9	3	6	41	4916
Scenario C	7	4	3	27.7	3326

## Technical Validation

The following steps were taken to ensure data quality during the experimental campaigns.

### Sensor calibration

All temperature, level, and flow sensors were calibrated using standard calibration protocols specified by the equipment manufacturers (Table 2). Sensor drift and failure were monitored, and affected instruments were corrected or replaced to avoid erroneous measurements. GC methods for analysis were calibrated with pure components, and measurement accuracy was assessed with test samples of known composition. Karl Fischer titration was calibrated with a standard coulometric solution, and accuracy was assessed with known samples.

#### Aiming at steady-state operation

The experiments were conducted with the aim of reaching steady-state operation. The startup and shutdown phases were trimmed, and the retained intervals were selected once the main process variables showed no systematic trend in their 30-min moving averages. No single numerical tolerance was applied across all runs because the variability expected at a given operating point depended on the chemical system, feed rate, and control structure. The acceptable variation was therefore assessed separately for each scenario and process variable. For the n-butanol–water system, for example, temperatures generally remained within approximately 0.3–0.7 K of their operating levels.

Flow rates showed considerably greater variability, particularly because the level controller maintained the reboiler level by adjusting the bottoms flow. Consequently, the bottoms flow absorbed process variability even when the main process variables remained stable. Mass-flow signals derived from weighing-scale measurements also contained occasional spikes caused by short-term instability of the scale readings. These spikes were regarded as part of the measurement behavior during normal operation but were excluded when calculating mean flow rates and other summary statistics. The retained intervals, therefore, represent practical steady-state operation, apart from routine disturbances, measurement variability, and changes caused by controller actions. The assessment was made separately for each run based on the expected process behavior, thermodynamics, and operating experience.

#### Comprehensive variable logging

The data collected include both manipulated variables and system responses. The raw process data include column temperatures, mass flow rates, thermostat temperatures, column head pressure, pump settings, pressure differential indicators, actuator commands, and analytical data derived from gas chromatography and Karl Fischer titration.

#### Overall material balance

The overall material balance was evaluated for sample runs. Losses relative to the feed were 7.9% in the water runs and 6.4% in the runs with n-butanol–water. Losses of this magnitude are expected at this scale, given the withdrawal of liquid samples during an experiment, the collection of uncondensed vapor in the cold traps, and hold-up in the buffer vessels. For more details regarding OME runs, refer to the publication by Ferre *et al*.^35^.

#### Repeatability and metadata documentation

Multiple experimental runs were conducted at each operating condition. A detailed log was maintained documenting the column configuration, the time required under constant operating conditions, feed composition, column startup and shutdown, any deviations or anomalies, and the steps taken to fix the anomaly. This metadata from each experiment was also published along with the data.

#### Identification of anomalies

Not all deviations from signals that remain approximately stable during normal operation can be classified as an anomaly. For example, certain process variables, such as mass flow rates measured with weighing scales, may exhibit spikes and outliers that cannot be classified as anomalies, as this is expected normal behavior. For manually induced anomalies, the operator is aware of the affected sensor and the time of occurrence. Naturally occurring anomalies, on the other hand, were identified through visual inspection of the data, and any unusual outliers or abnormal behavior patterns were labeled as anomalies based on the accepted threshold of the process variables, chosen based on the behavior expected under constant operating conditions at the corresponding operating point, considering the system thermodynamics, operating parameters, and the operator’s practical experience. The identified anomalies were labeled in the final dataset in separate columns. A detailed description of the anomaly labels is provided in the Data Records section. Anomaly details specific to each scenario are also provided in the metadata files, including the anomaly’s start and end times, the affected sensors, and its cause. This metadata scheme is explained in detail in our related work^31^.

#### Accurate data labels

Windows of constant operating conditions and labels distinguishing normal process data from anomalies are defined differently for naturally occurring anomalies vs. manually induced anomalies. The labeled experimental data are then split into training runs (without anomalies) and test runs (with anomalies). Further details of the headers in the data files are provided in the Data Records section.

### Sample data without anomalies

**Fig. 5:**
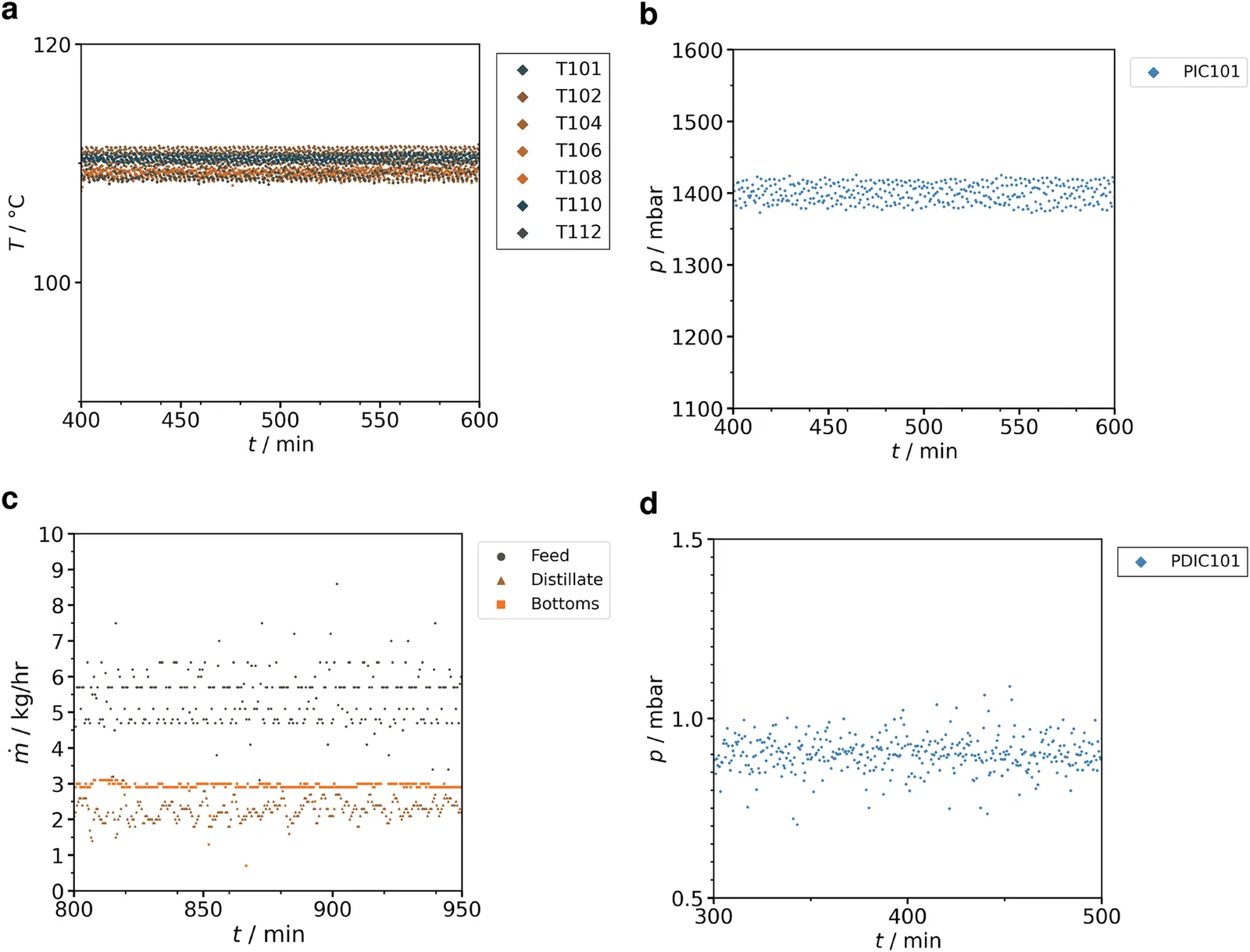
Sample operating data with water during normal operation. **a,** Column temperatures. **b**, Column head pressure. **c**, Mass flow rates around column 1. **d**, Reboiler differential pressure. Panels **a,** **b**, and **d** show train_normal_experiment_001 at operating point 007; panel **c** shows train_normal_experiment_001 at operating point 004.

**Fig. 6:**
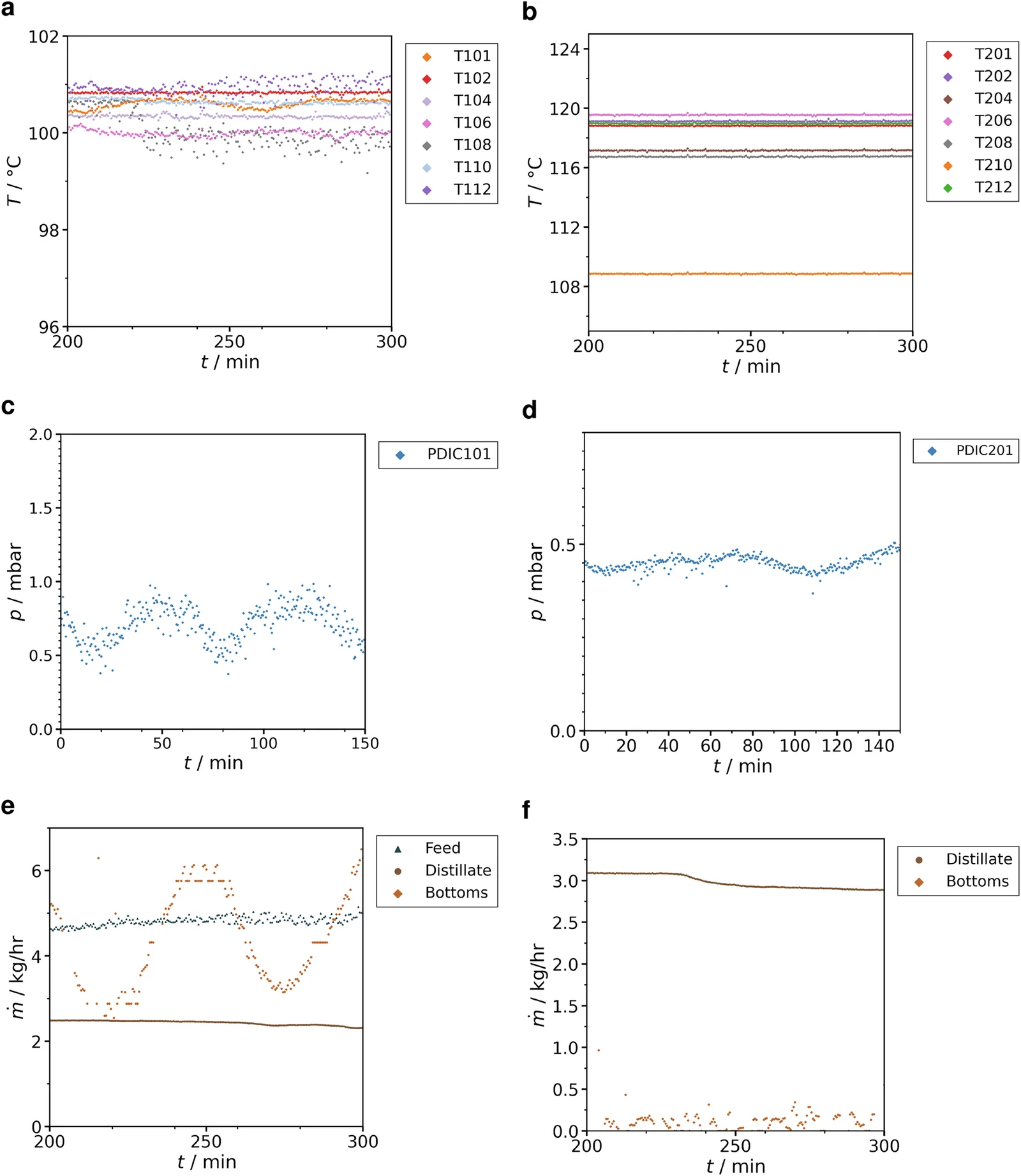
Sample operating data with n-butanol–water during normal operation. **a**, Temperatures in column 1. **b**, Temperatures in column 2. **c**, Reboiler differential pressure in column 1. **d**, Reboiler differential pressure in column 2. **e**, Mass flow rates around column 1. **f**, Mass flow rates around column 2. All panels show train_normal_experiment_001 at operating point 006.

**Fig. 7:**
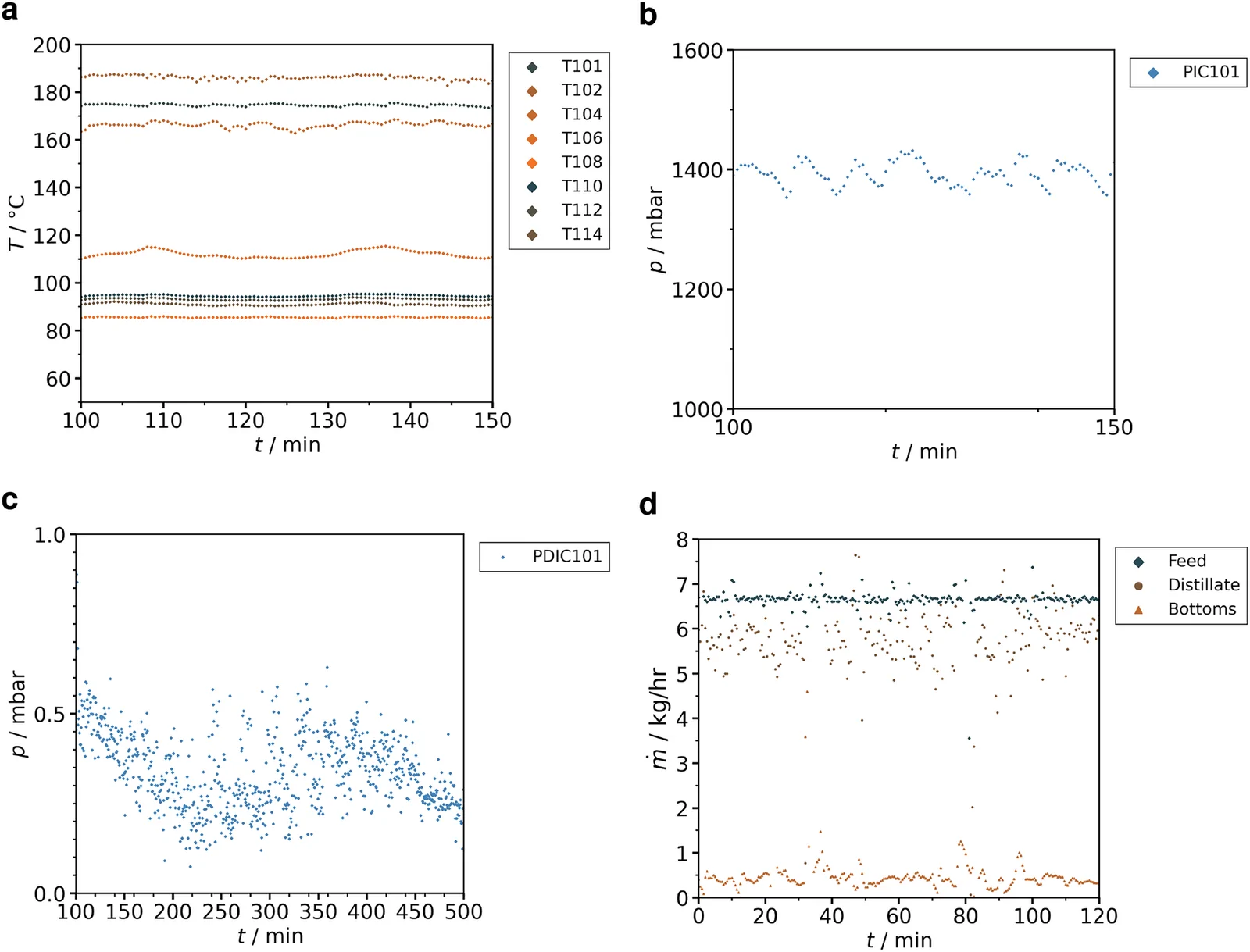
Sample operating data with OME during normal operation. **a**, Temperatures in column 1. **b**, Column head pressure. **c**, Reboiler differential pressure. **d**, Mass flow rates around column 1. Panels **a** and **b** show train_normal_experiment_001 at operating point 002; panels **c** and **d** show train_normal_experiment_001 at operating point 001.

Figures 5, 6, and 7 show the temperature profiles in the columns, the differential pressures in the reboilers as a measure of their level, and the mass flow rates to and from the columns, during the fault-free operation in Scenarios A, B, and C, respectively.

#### Water runs

Figure 5a shows that all column temperatures in the water runs lie in a narrow band close to 108 ^∘^C and are nearly constant with time. Water is a single component, so no composition gradient can develop along the column and no temperature profile is expected; Fig. 5b shows the head pressure. It stays close to 1.4 bar (a) throughout, varying within a band of roughly  ±20 mbar. The pressure was held by feeding a small flow of inert gas into the column head, and this flow could not be metered finely enough to hold the pressure at a single value. The width of the band is therefore set by how precisely the control can act, and the scatter does not indicate a change of operating point. Figure 5c shows the feed, distillate, and bottoms flow rates for a sample water run. The bottoms flow is comparatively smooth, while the feed and distillate traces contain spikes which occur throughout normal operation and are not anomalies. The differential pressure in the reboiler, Fig. 5d, fluctuates about 0.9 mbar as the level controller acts.

#### N-butanol–water runs

The temperatures in Fig. 6a,b are nearly constant, except for T108 at the feed stage and T101 in the reboiler. T108 responds to disturbances in the incoming feed, and T101 varies with the reboiler level because changes in liquid level affect the immersion of the temperature sensor and, consequently, the measured temperature. Fig. 6c,d show the differential pressures across the reboilers (PDIC101, PDIC201), which we used as a measure of the reboiler levels. A P-only controller held the level by adjusting the power of the bottoms pump (P102, P202). The feed (F104), bottoms (M103, M203), and distillate (F102, F103) flow rates are shown in Fig. 6e,f. The bottoms flows fluctuate noticeably, but this is how the loop is meant to work. The feed was delivered by a membrane pump with a manual valve and was not under flow control, so slow changes in the feed rate reached the reboiler level and, through the level controller, the bottoms flow. The controller shifted the variability from the level to the outflow, keeping the level within its operating window. The magnitude of the fluctuation depends on the controller gain, which we set to keep the level within its limits; a lower gain would have resulted in smoother outflow and larger level swings. Normal operation is therefore hard to recognize from any single signal. The temperatures are steady, while the flow rates show spikes, drifts, and slow variations that all belong to normal operation.

#### OME runs

The OME runs in Fig. 7a show the opposite picture. Temperatures range from about 85 ^°^C at the top of the column to about 190 ^°^C in the reboiler and increase monotonically towards the bottom, as expected for a mixture whose components differ widely in boiling temperature. Each sensor is stable in time, and the separation between adjacent sensors reflects the composition profile rather than measurement scatter. The head pressure, Fig. 7b, is held at about 1.4 bar (a) and shows a slow periodic variation of a few minutes, following the action of the pressure controller. The differential pressure in the reboiler, Fig. 7c, shows a similar pattern to the n-butanol–water system. Figure 7d shows a steady feed flow of approximately 6.7 kg h^−1^ and a bottoms flow of a few tenths of a kilogram per hour, consistent with OME_3–5_ constituting a relatively small, higher-boiling product fraction of the feed.

### Sample data with anomalies

#### Water runs

**Fig. 8:**
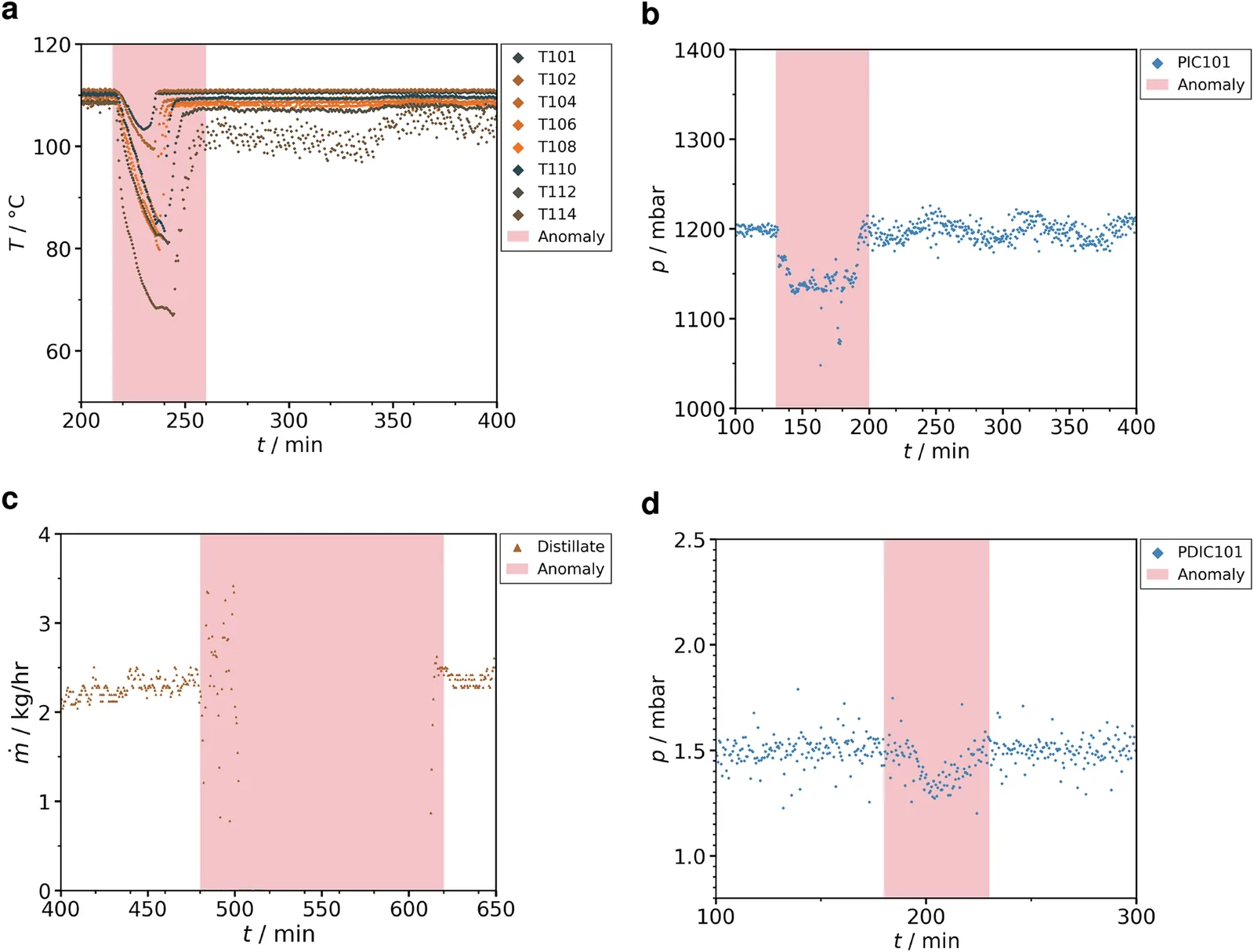
Sample time-series profiles from anomalous water experiments in column 1. **a**, Column temperatures. **b**, Column head pressure. **c**, Distillate mass flow rate. **d**, Reboiler differential pressure. Panels **a** and **b** show test_anormal_experiment_001 at operating point 001; panel **c** shows test_anormal_experiment_001 at operating point 005; panel **d** shows test_anormal_experiment_001 at operating point 007.

Figure 8a shows anomalous runs with water. The column temperatures drop sharply from their normal band near 108 ^°^C, the lower stages falling to below 70 ^°^C, and recover once the cause is removed. The sensors nearest the reboiler deviate most and return last, which is consistent with a loss of boil-up propagating upwards through the column. The head pressure in Fig. 8b falls by about 80 mbar before returning to its previous level. In Fig. 8c, the distillate flow rate becomes anomalous during the annotated period, swinging between zero and roughly 3.5 kg h^−1^, in contrast to the rather steady value of about 2.3 kg h^−1^ before and after. The reboiler differential pressure, Fig. 8d, dips by around 0.2 mbar during the anomaly and recovers afterward. In each case shown here, the affected variable returns to its previous value once the cause is removed, which distinguishes these anomalies from the drifts described above. In the water system, the most frequently encountered anomalies were unstable reboiler levels, reboiler overheating, feed-line clogging, distillate-line clogging, temperature-sensor issues, head-pressure-sensor issues, and faulty instruments.

#### N-butanol–water runs

**Fig. 9:**
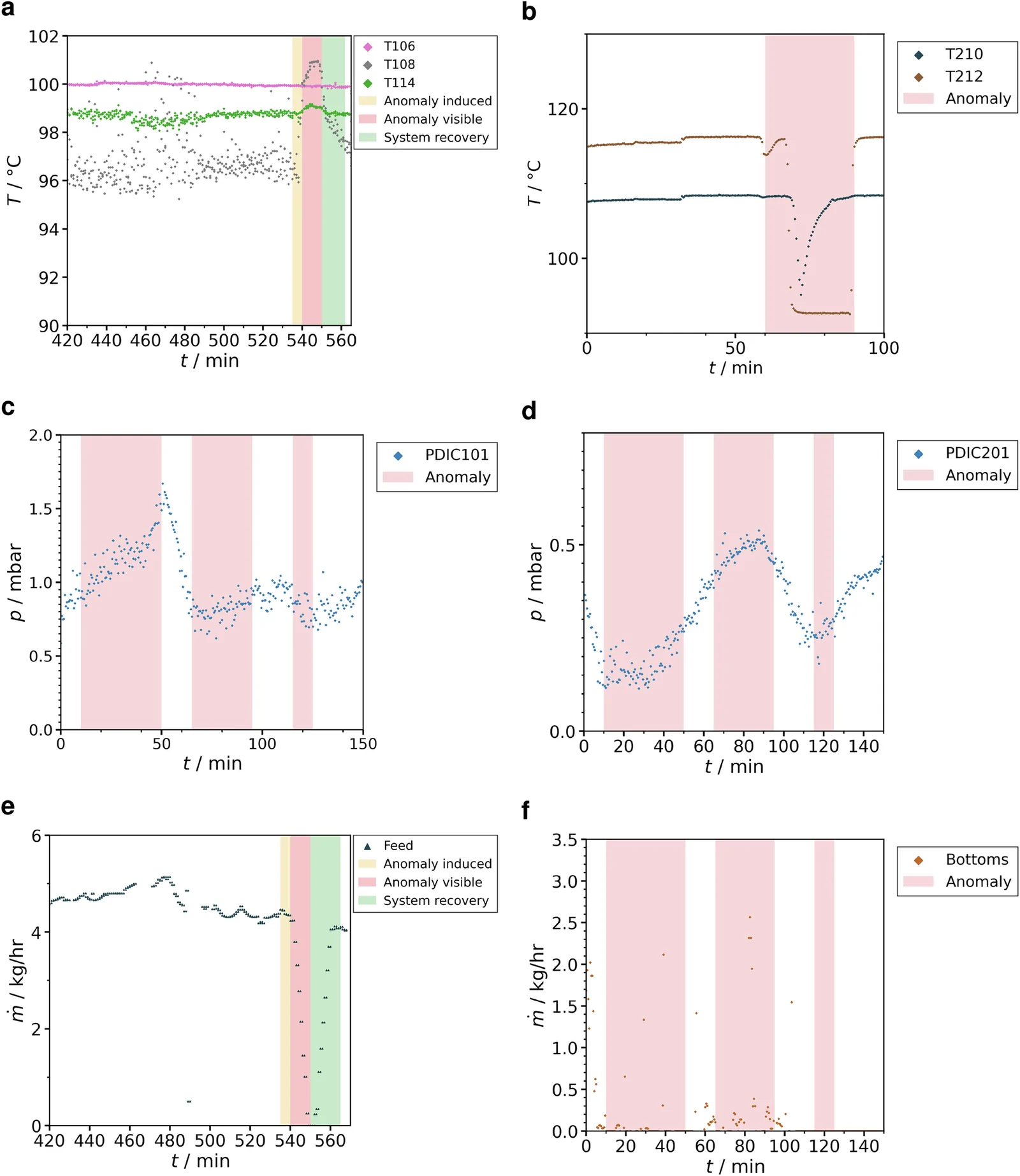
Sample operating data from Scenario B with n-butanol–water during anomalous operation. **a**, Temperatures in column 1. **b**, Temperatures in column 2. **c**, Reboiler differential pressure in column 1. **d**, Reboiler differential pressure in column 2. **e**, Feed mass flow rate to column 1. **f**, Bottoms mass flow rate from column 2. Panels **a** and **e** show test_anormal_experiment_001 at operating point 005; panel **b** shows test_anormal_experiment_001 at operating point 001; panels **c,** **d**, and **f** show test_anormal_experiment_002 at operating point 001.

Figure 9a,b represent anomalous temperatures. Spikes can be observed in T108 in Fig. 9a, due to manually triggered clogging in the feed line. A reboiler level anomaly could lead to flooding or column drying if not identified promptly. Figure 9c,d show reboiler levels during an anomalous run with the occurrence of three anomalies (a level sensor anomaly in C2, a temperature sensor anomaly in C1, and a feed line clogging anomaly in C1). Figure 9e shows the feed mass flow rate that was affected due to a manually induced pipeline clogging via a hand valve in the feed pipeline to C1. Figure 9f, shows that the C2 bottoms flow remained close to zero during the simultaneous anomaly events.

#### OME runs

**Fig. 10:**
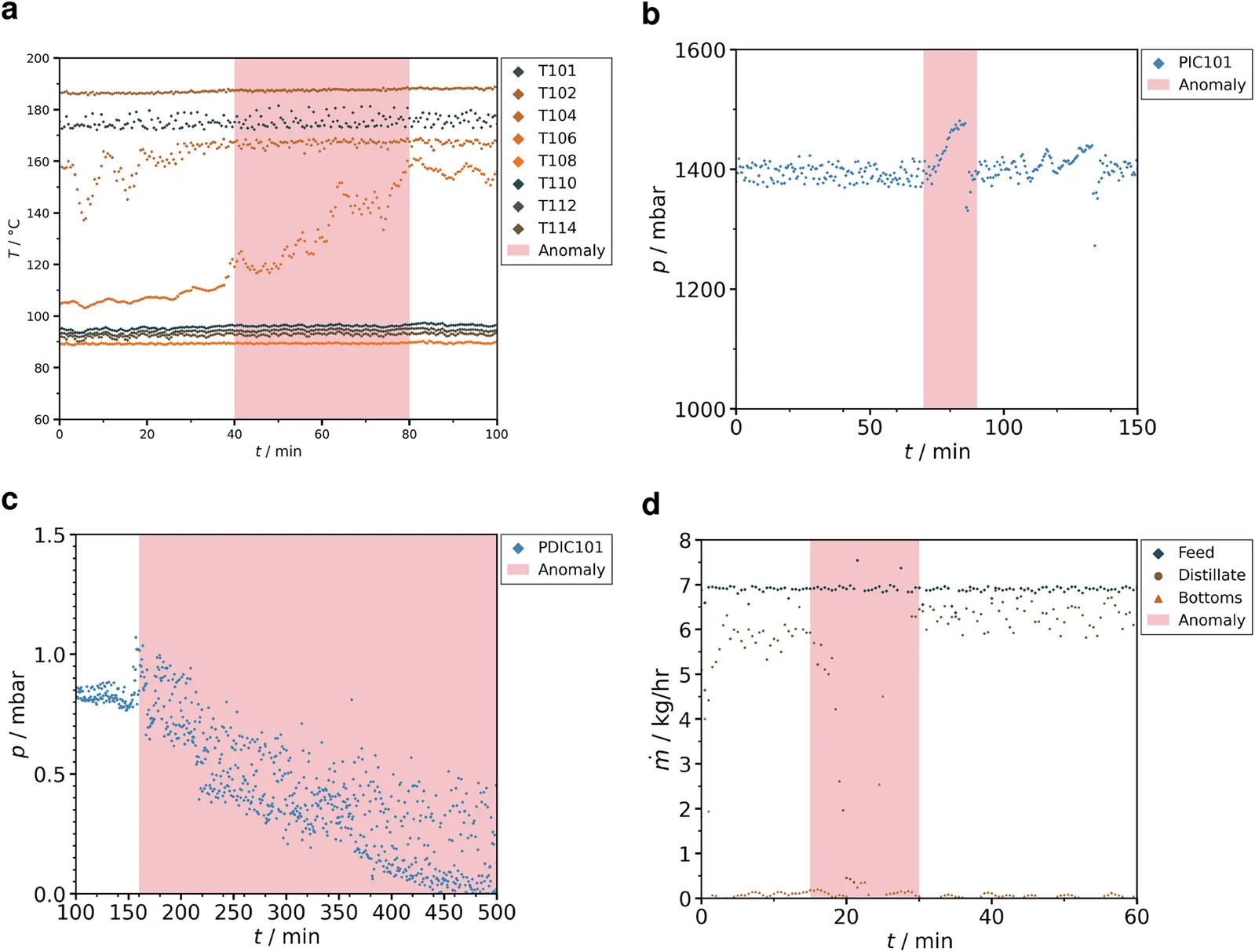
Sample operating data with OME during anomalous operation. **a**, Column temperatures. **b**, Column head pressure. **c**, Reboiler differential pressure. **d**, Mass flow rates around column 1. Panels **a** and **d** show test_anormal_experiment_001 at operating point 004; panels **b** and **c** show test_anormal_experiment_001 at operating point 007.

Figure 10a shows an anomalous run with OME. Most temperatures remain at their normal values, but the sensor in the middle of the column rises from about 110 ^°^C to above 140 ^°^C during the annotated period and falls back afterwards, while the sensors above and below it are barely affected. The head pressure in Fig. 10b rises by about 100 mbar during a shorter annotated period. Figure 10c shows the reboiler differential pressure falling from about 0.9 mbar towards zero over the course of the run and remaining there, so the effect of this anomaly persists rather than recovering. In Fig. 10d, the feed flow rate is disturbed during the annotated period while the bottoms flow, which is small throughout, changes little. For the OME system, anomalies were encountered with temperature sensors, reboiler level, and faulty instruments.

## Usage Notes

The dataset is intended primarily for developing and testing anomaly detection methods. Runs with and without anomalies are labeled and provided separately, so the data can be used with supervised, semi-supervised, or unsupervised methods. We have used the entire dataset to compare methods from the literature^20,32^ and to test time-series AD benchmarking libraries^33^. The data can also be used to train generative models for synthetic process data^42–45^. Purely data-driven models tend to be unreliable outside the operating points they were trained on, so a mechanistic model of the plant calibrated against these data is likely to be the better route. We plan to build such a model. Since many variables were recorded simultaneously over long periods, the data are well-suited to multivariate visualization^46–48^, including dimensionality reduction and interactive plotting to explore relationships among sensors and explain anomalies.

All data are recorded at 30 s intervals. In Scenario B, the two columns were logged by separate acquisition systems that could not be synchronized during the experiments, so their timestamps were aligned onto a common 30 s grid afterward. Readings from the two columns should therefore be treated as near-simultaneous rather than exactly so. This does not apply to Scenarios A and C, where a single system was used, and the recorded timestamps are unchanged.

File names mark which runs contain anomalies, not a split we recommend. Users are free to divide the runs however they like, and for methods that require anomalies during training, the labeled test runs can be used for that purpose. The labels distinguish induced from naturally occurring anomalies: naturally occurring ones are labeled 0 or 1, while induced ones also indicate the period between the anomaly’s induction and its first appearance in the data, as well as the recovery afterward. Methods that only need a binary label can treat 1, 2, and 3 together.

The anomalies in this dataset are a subset of those in Kister’s survey of tower malfunctions^5^. The dataset covers clogging of the feed, distillate and bottoms, reboiler level instabilities; reboiler overheating, condenser control problems, and coolant failures; faulty level and temperature measurements; pressure control failures; and operator and control system errors. It does not cover faults that require long operating times or industrial scale conditions to develop: coking, corrosion, fouling of the internals, mechanical damage to the packing, foaming, leaks, and maldistribution in wide columns. Every anomaly here develops over minutes to hours, so slow degradation is absent, and the plant is small enough that scale-dependent effects cannot appear.

The dataset, therefore, samples only one part of the range of faults that occur in a distillation column, and results obtained on it should not be assumed to generalize to the rest. This publication focuses on operation under constant operating conditions. Startup and shutdown were therefore removed from the records. Work on transient operation is ongoing within our project group, and we intend to release the corresponding transient-operation data in future work. Meanwhile, transient behavior is covered by our companion dataset on batch distillation^31^, where every run is a transient.

A web application at https://continuousdistillationtum.streamlit.app/ allows visualization of the data without downloading it. Any run from the three scenarios can be selected by operating point and experiment index, and its metadata is displayed alongside the data. Variables can be plotted individually or together, as time series, rolling averages, heatmaps, autocorrelations, or seasonal decompositions, and the annotated periods are shaded in runs that contain anomalies. For the runs with n-butanol–water, the measured concentration profiles are also available. The application is intended for a first inspection of the data, for instance, to choose which runs to work with; the deposited files remain the primary source.

## Supplementary information

Supplementary Table 1

## Data Availability

The time-series data described in this work are available in a publicly accessible repository from Zenodo^40^. This repository has a structure and data file format as described in the Data Records section.

## Code availability

Two custom scripts were used in this work, both written in Python. The raw records were processed with a script that trimmed the startup and shutdown phases, aligned the timestamps of the two data acquisition systems used in Scenario B onto a common 30 s grid, and averaged readings recorded at 20 s intervals within each window. It is available at https://github.com/aparnamuraleedharan/DataProcessing (release v1.0, archived at https://doi.org/10.5281/zenodo.21814763) and depends on pandas and NumPy.

The web application used to visualize the data was built with Streamlit. Its source code is available at https://github.com/aparnamuraleedharan/datavisualization (release v1.0.1, archived at https://doi.org/10.5281/zenodo.21815051). Both repositories are released under the MIT license. LabVIEW v.19 (National Instruments) was used to operate the plant and record the raw data.
